# Intrahepatic cholangiocarcinoma: Insights on molecular testing, targeted therapies, and future directions from a multidisciplinary panel

**DOI:** 10.1097/HC9.0000000000000743

**Published:** 2025-06-09

**Authors:** Rushabh Gujarathi, Supriya Peshin, Xuchen Zhang, Melinda Bachini, Molly N. Meeks, Rachna T. Shroff, Anjana Pillai

**Affiliations:** 1 Department of Medicine, University of Chicago Medicine, Chicago, Illinois, USA; 2 Department of Internal Medicine, Norton Community Hospital, Norton, Virginia, USA; 3 Department of Pathology, Yale School of Medicine, New Haven, Connecticut, USA; 4 Cholangiocarcinoma Foundation, Herriman, Utah, USA; 5 Department of Medicine, University of Arizona Cancer Center, Tucson, Arizona, USA

**Keywords:** biliary tract cancers, cholangiocarcinoma, liver transplantation, molecular testing, targeted therapies

## Abstract

Biliary tract cancers (BTCs) are a histologically and molecularly diverse group of malignancies arising from the gallbladder and the ductal epithelium of the biliary tree. Intrahepatic cholangiocarcinoma (iCCA) is the second most common primary liver malignancy in the United States. Surgical resection with negative margins is the only recognized curative treatment option for iCCA; however, most patients will present with advanced or unresectable disease. The clinical presentation is largely non-specific, with the characteristic symptoms of biliary malignancies being less frequent than extrahepatic cholangiocarcinoma. Clinical management in iCCA is heavily influenced by the molecular profile of individual tumors. Hence, pathologists must exercise caution to prevent tissue exhaustion during the diagnostic workup of iCCA and ensure the availability of tissue samples for molecular testing. Establishing standardized procedures for obtaining adequate tissue and using molecular testing is vital. Circulating tumor DNA (ctDNA) offers a potential alternative to tissue-based analysis, especially in cases with insufficient tissue samples. Drugs targeting alterations in *NTRK*, *IDH1*, *BRAF*, *FGFR2*, and *HER2* are commonly utilized. Targeting the MDM2–p53 pathway represents an avenue for future investigations in advanced BTCs. Liver transplantation and locoregional therapies are treatment modalities that may represent curative intent treatments for patients with unresectable disease, and larger explorations are warranted. Akin to HCC, a multidisciplinary team–based approach is essential for patients with BTCs. Through this narrative review of literature, we provide an overview of the current management of iCCA with perspectives regarding future directions in the clinical management of iCCA. We also present patient perspectives regarding the importance of patient advocacy and access to advances in clinical research for patients with BTCs.

## INTRODUCTION

Biliary tract cancers (BTCs) encompass a range of rare yet highly aggressive group of malignancies, typically adenocarcinomas, which originate from the gallbladder/cystic duct (gallbladder carcinoma) or from the ductal epithelium of the biliary tree (cholangiocarcinomas).^1^,^2^

**FIGURE 1:**
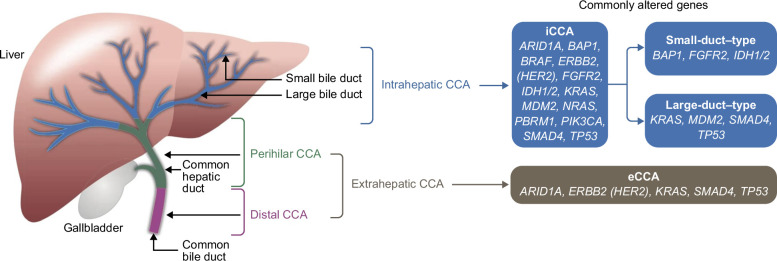
Anatomic classification of cholangiocarcinoma and common target genes altered in cholangiocarcinoma subtypes. Abbreviations: CCA, cholangiocarcinoma; eCCA, extrahepatic cholangiocarcinoma; iCCA, intrahepatic cholangiocarcinoma. Reprinted from Gopal et al^4^ with permission from *Archives of Pathology & Laboratory Medicine*. Copyright 2024. College of American Pathologists.

Cholangiocarcinomas (CCAs) can be further classified according to their anatomical site of origin into intrahepatic cholangiocarcinoma (iCCA), which develops within the intrahepatic bile duct epithelium, and extrahepatic cholangiocarcinoma (eCCA). The latter is further subdivided into perihilar CCA (pCCA) and distal CCA (dCCA). The second-order bile ducts serve as the anatomical point of separation between iCCA and eCCA, with all CCA arising proximal to (and including) the second-order or segmental bile ducts being termed iCCA.^1^,^2^ The pathobiology of iCCA differs from that of eCCA in various aspects, notably their molecular biology, which results in differences in their clinical management (Figure 1).^3^,^4^

Cholangiocarcinomas comprise a diverse group of BTCs known for their dismal prognosis, primarily due to their diagnosis at advanced stages and aggressive biological behavior. The Surveillance, Epidemiology, and End Results (SEER) database underscores the severity of this issue, revealing that most iCCA patients are diagnosed at advanced tumor stages, with 20.1% presenting with stage III and 34.9% with stage IV disease.^5^ The incidence trends of iCCA in the United States are indicative of a growing oncologic challenge. As per recent data from the SEER registry, iCCA is the second most common primary liver cancer in the United States, with a recorded incidence of 1.19 per 100,000 person-years in the period between 2001 and 2017.^6^ The annual percentage change in the incidence of iCCA showed significant increase from 2007 to 2012, as well as from 2013 to 2017.^6^ Projections based on SEER data also suggest that the incidence of iCCA in 2029 will be nearly double what it was in 2001, with an increase in incidence up to 2.13 cases per 100,000 individuals being projected.^7^ Although SEER data have shown a decline in iCCA mortality from 2001 to 2016,^6^ reports from the National Center for Health Statistics Database are conflicting in this regard.^8^ As separate International Classification of Diseases (ICD) codes for recording iCCA and the subtypes of eCCA were introduced for the first time in 2021, epidemiological trends from previously reported data should be interpreted with caution.^9^

Intrahepatic cholangiocarcinomas pose substantial challenges in diagnosis and treatment. The clinical presentation of iCCA is usually non-specific, with abdominal pain being the most common presenting symptom.^10^ Jaundice and weight loss are far less common than eCCA and are seen in less than 20% of patients with iCCA at presentation.^10^,^11^ The non-specific presentation is also exemplified by the proportion of patients having surgically unresectable disease at presentation. Only 20%–30% of patients with iCCA present with disease that is considered amenable to curative surgical resection.^12^

Through this narrative review, we provide current perspectives regarding the clinical management and treatment of iCCA, highlighting the importance of molecular testing in determining treatment decisions and redefining curative options, including the important role of patient advocacy, clinical trials and multidisciplinary team input in optimizing individualized patient care.

## DIAGNOSIS AND MOLECULAR TESTING OF ICCA

Advances in high-throughput molecular techniques, particularly next-generation sequencing (NGS), have improved our understanding of CCA, revealing targetable genetic alterations in 40%–50% of patients.^13^,^14^,^15^ The use of targeted therapies relies on an accurate pathologic diagnosis, but diagnosing CCA remains challenging due to the lack of specific immunohistochemical markers and the high frequency of metastasis to the liver.

### Integrating molecular testing into clinical practice to inform treatment decisions

Once a tumor is sampled via tissue core needle biopsy (CNB) or fine needle aspiration (FNA), the main challenge in diagnosing CCA is distinguishing it from other hepatic lesions, such as HCC and metastatic adenocarcinoma to the liver or biliary tree. Differentiating CCA from metastatic adenocarcinoma can be challenging due to similarities in clinical presentation, overlapping histopathologic features, and the absence of CCA-specific tissue markers.^4^ While distinguishing CCA from HCC is usually straightforward, in more complex cases, further immunohistochemical markers are required to make the distinction.^4^ A panel of immunohistochemical markers is commonly used to confirm iCCA diagnosis, classify its subtypes, and differentiate it from metastatic adenocarcinoma or HCC. However, pathologists must exercise caution when selecting the minimal number of stains necessary, especially given the limited tissue obtained from CNB or FNA. In the era of precision medicine, preserving tissue for potential biomarker and genomic analyses is crucial, and immunohistochemical markers should be employed in a stepwise manner.^16^ Additionally, to prevent tissue exhaustion before biomarker analysis, it is recommended that CNB specimens be split into 2 blocks: one for diagnosis and immunohistochemical stains, and the other for biomarker analysis.^17^

Once diagnosed, iCCA may be categorized into small duct and large duct types based on histopathological criteria. Small duct iCCA predominantly displays tubular structures with definitive lumina formed by cuboidal to low columnar tumor cells, nestled within a desmoplastic stroma. In contrast, large duct type iCCA often resembles pCCA and dCCA with papillary and glandular architecture and is often preceded by precancerous lesions like biliary intraepithelial neoplasia or intraductal papillary neoplasia. Mucin secretion is common in large duct iCCA but usually absent in small duct iCCA. Genomic alterations correlate with histopathological features: small duct iCCA more frequently exhibits *IDH1/2* mutations and *FGFR2* fusions and has a lower incidence of *KRAS* mutations. In contrast, large duct iCCA shares genetic profiles with pCCA and dCCA, showing high frequencies of *KRAS*, *TP53*, *SMAD4*, *ERBB2* (*HER2*), and *MDM2* mutations, along with *ERBB2 (HER2)* amplification. Silencing mutations in chromatin remodeling genes such as *PBRM1*, *ARID1A*, and *BAP1* are seen in CCA. Additionally, rarer genetic alterations, including *NTRK1/2/3*, *ROS1*, *ALK* fusions, *BRAF* mutations, and microsatellite instability−high (MSI-H)/mismatch repair deficiency (dMMR), have also been reported in CCA.^4^,^18^,^19^

The National Comprehensive Cancer Network (NCCN) and European Society for Medical Oncology (ESMO) guidelines recommend comprehensive molecular profiling for patients with unresectable or metastatic BTC, which helps identify potential therapeutic targets. If tissue is insufficient or unavailable, repeat biopsy may be considered based on tumor accessibility, safety, and clinical context.^20^,^21^ These genetic alterations not only aid in diagnosing CCA but also guide targeted therapeutic decisions. Genetic alterations may also help predict responses to systemic therapy.^22^,^23^ Targeted therapies approved by the U.S. FDA and European Medicines Agency (EMA), or under clinical investigation, include FGFR2 inhibitors in tumors with *FGFR2* fusions, IDH inhibitors in tumors with *IDH1/2* mutations, RAS–BRAF–MEK–ERK pathway inhibitors in tumors with *BRAF* mutations, HER2 inhibitors in tumors with *ERBB2* amplifications, NTRK inhibitors in tumors with *NTRK* alterations, and immune therapy for MSI-H/dMMR tumors.^4^,^18^,^19^,^21^ A study found that chemotherapy responses were more favorable in small duct type iCCA with *BAP1* and *IDH1* mutations compared with large duct type iCCA with *KRAS* and *SMAD4* mutations.^23^ Such targeted approaches have shown promising results in clinical trials, offering new hope for patients with advanced CCA.

### Currently available molecular testing methods and limitations

The molecular characterization of CCA has revolutionized its diagnosis and treatment. Multiple molecular testing methodologies are now in use, each bearing specific benefits and limitations. Next-generation sequencing (NGS) is the most widely used method, enabling comprehensive genomic profiling by identifying mutations, fusions, and copy number alterations. This approach provides in-depth insights into the genomic landscape of CCA, aiding in the development of personalized treatment strategies. However, NGS is not without its drawbacks, such as the need for high tumor content from the tissue for accurate analysis, high costs, and the requirement for specialized equipment and expertise. In Situ Hybridization (ISH) based assays can detect gene fusions and amplifications (eg, *FGFR2* fusion, *HER2* amplification) with high specificity, but are limited by the number of targets they can assess simultaneously. PCR is useful for detecting specific known mutations and MSI status, but with the limitation of its inability to discover novel alterations. Immunohistochemistry (IHC) can identify protein expression patterns indicative of underlying genetic changes (eg, *HER2* expression and MMR status), but its qualitative nature can lead to variability in interpretation. These methodologies each have roles depending on the clinical scenario. IHC is widely used in pathology laboratories for diagnostic purposes and biomarker detection. Reflex testing of biomarkers like MMR and HER2 can begin immediately after a pathological diagnosis, rather than waiting for a request later from an oncologist. This approach reduces delays from diagnosis to the delivery of clinically important biomarker results. It can also help ensure timely testing for all patients with CCA. However, reflex testing has drawbacks. It may increase costs, particularly when clinical details like tumor stage are unavailable to pathologists, leading to unnecessary testing in early-stage cases where targeted therapy is not needed. Additionally, the rising use of NGS may render some IHC tests redundant. NGS offers a comprehensive view of genomic alterations, making it increasingly preferred over single-marker tests in CCA and potentially more cost-effective.

A major challenge in biomarker testing for CCA is obtaining sufficient tissue. This difficulty is partly due to the tumors’ challenging location, as well as the presence of desmoplastic stroma and tumor necrosis, which can reduce the tumor cell content in biopsy samples. In an analysis of 123 tissue samples from patients with advanced BTC (68.2% iCCA), 26.8% failed NGS analysis, primarily due to insufficient tumor content (<20%).^24^ FNA samples generally have better cellularity and higher tumor fractions than CNB samples, making FNA more suitable for routine molecular diagnostics. FNA can be integrated into the molecular diagnostics workflow alongside CNB samples to maximize the use of limited tissue for clinically relevant genomic testing.^25^ To improve success rates, concurrent CNB and FNA have been used in clinical trials,^26^,^27^ though this practice has not been standardized, highlighting the need for further research across different tumor types, including CCA.^17^,^28^ Therefore, establishing standardized procedures for obtaining adequate tissue and using molecular testing across institutions is essential. Integrating findings from molecular biology, genomics, and clinical trials into patient management protocols will likely improve outcomes and offer new avenues for therapeutic development.

When tissue samples are insufficient and/or unavailable, circulating tumor DNA (ctDNA) may offer a potential alternative to tissue-based analysis. A recent large-scale study of liquid biopsy focusing on ctDNA showed good concordance with tissue analysis for identifying therapeutic targets and actionable genetic alterations in advanced BTC.^29^ Additionally, an extended analysis of the STAMP trial found that ctDNA status and dynamics could predict recurrence during adjuvant therapy, helping to optimize clinical decision making and treatment plans in resected eCCA.^30^ Despite these advances, variability in liquid biopsy collection and analysis methods has prevented its standard clinical use.^4^ Furthermore, the complexity of CCA necessitates a multidisciplinary approach encompassing pathologists, oncologists, radiologists, geneticists, and researchers working collaboratively to navigate the diagnostic challenges and innovate treatment strategies.

## TREATMENT OPTIONS FOR ICCA

### Frontline systemic treatment for advanced disease

**FIGURE 2:**
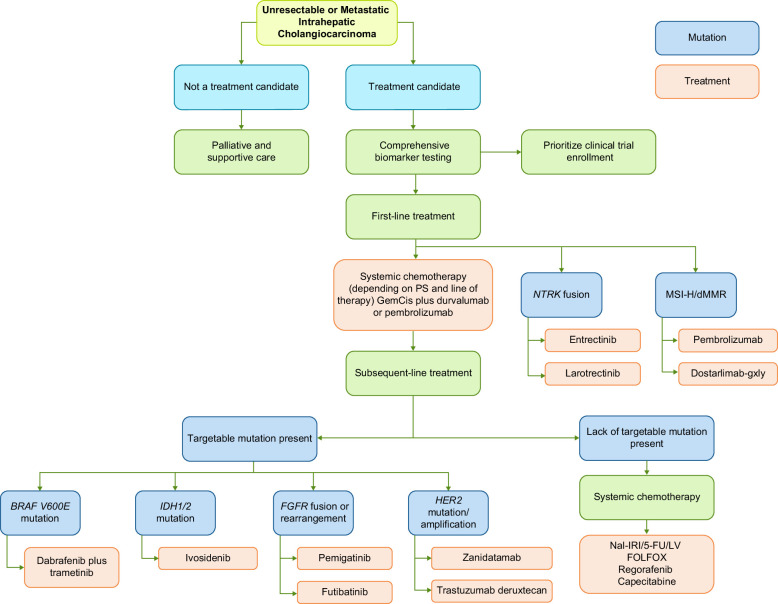
A comprehensive approach to systemic therapies in intrahepatic cholangiocarcinoma. Abbreviations: dMMR, mismatch repair deficiency; FOLFOX, leucovorin/fluorouracil/oxaliplatin; GemCis, gemcitabine/cisplatin; MSI-H, microsatellite instability—high; Nal-IRI/FU/LV, liposomal irinotecan/fluorouracil/leucovorin; NGS, next-generation sequencing; PS, performance status.

Despite advancements in therapeutic strategies, BTC remains a formidable challenge, with a 5-year survival rate of just 15%.^31^ The prognosis is particularly grim for patients with iCCA, which has a 5-year survival rate of only 8.5%, and for those with metastatic disease, where survival plummets to a mere 3%.^32^ For early-stage iCCA, surgical resection remains the only potentially curative option. Typically, surgery is combined with adjuvant capecitabine therapy, as recommended by the BILCAP trial.^33^ This trial demonstrated an improvement in recurrence-free survival (RFS), although no significant overall survival (OS) benefit was observed in the intent-to-treat population.^34^,^35^ Outcomes in early-stage iCCA vary, with 5-year survival rates ranging from 60% to 70%. However, factors such as lymph node involvement and R1 resections significantly diminish these rates.^36^,^37^ An R1 resection is conventionally defined as the presence of tumor cells at the liver transection line, indicating a microscopically positive surgical margin.^38^ Figure 2 presents a comprehensive framework outlining the approach to iCCA management.

### First-line treatment for advanced iCCA

Unfortunately, ~60%–70% of iCCAs are diagnosed at an advanced stage, necessitating systemic therapy.^39^ The combination of gemcitabine and cisplatin (GemCis) has been the cornerstone of first-line treatment for advanced iCCA. The landmark ABC-02 trial established its efficacy, demonstrating a median OS of 11.7 months compared to 8.1 months for gemcitabine monotherapy.^40^ Recent innovations in systemic therapy have included adding immunotherapy to a chemotherapy backbone. Durvalumab, an immune checkpoint inhibitor, has been incorporated into the GemCis regimen, offering a new standard of care based on the results of the TOPAZ-1 trial.^41^ Similarly, the KEYNOTE-966 study demonstrated a significant improvement in OS with pembrolizumab plus GemCis, compared with GemCis alone, and established pembrolizumab plus GemCis as a reasonable first-line regimen.^42^ Although these advancements are promising, additional efforts have explored triplet regimens to further improve outcomes. For example, SWOG-1815 (NCT03768414) evaluated the addition of nab-paclitaxel to GemCis, but this did not show significant OS improvements compared to GemCis alone.^43^,^44^ For patients whose disease progresses after first-line therapy, second-line treatment options are critical yet limited. Historically, second-line therapies have been associated with poor outcomes, with a median OS of 7.2 months and progression-free survival (PFS) of just 3.2 months.^44^,^45^ Among the chemotherapy options for patients without actionable mutations, FOLFOX (leucovorin/fluorouracil/oxaliplatin) is the standard of care. The ABC-06 trial highlighted an OS benefit of FOLFOX plus active symptom control (ASC) over ASC alone.^45^ Liposomal irinotecan (nal-IRI) combined with fluorouracil (5-FU) and leucovorin (LV) could be another viable option, with the most recent analysis of the NIFTY trial showing a PFS of 4.2 months with nal-IRI plus 5-FU/LV compared to 1.7 months for 5-FU/LV alone.^46^ However, results from the NALIRICC study, which included a larger proportion of patients with iCCA along with an ethnically dissimilar cohort, must also be taken into account when considering this regimen. In the NALIRICC study, the addition of nal-IRI to 5-FU plus LV did not improve PFS (2.6 mo vs. 2.3 mo) or OS (6.9 mo vs. 8.2 mo) and was associated with higher toxicity compared with 5-FU plus LV alone^47^ (Table 1).

**TABLE 1:** Selected key clinical trials in advanced biliary tract cancer

Trial name	Treatment evaluated	Phase	Population	Outcome	Significance
ABC-02	Gemcitabine+cisplatin vs. gemcitabine alone^39^	Phase 3	Advanced biliary tract cancers	Improved median OS (11.7 vs. 8.1 mo)	Established gemcitabine+cisplatin as the standard of care for advanced BTC
TOPAZ-1	Durvalumab+gemcitabine/cisplatin vs. placebo+gemcitabine/cisplatin^41^	Phase 3	Advanced biliary tract cancers	Improved OS (12.8 vs. 11.5 mo)	Immunotherapy (durvalumab) was introduced as a first-line treatment for BTC
KEYNOTE-966	Pembrolizumab+gemcitabine/cisplatin vs. placebo+gemcitabine/cisplatin^42^	Phase 3	Advanced or unresectable biliary tract cancers	Improved median OS (12.7 vs. 10.9 mo); HR=0.83 (95% CI, 0.72–0.95); *p*=0.0034	Demonstrated that adding pembrolizumab to standard chemotherapy provides a statistically significant and clinically meaningful improvement in OS for patients with advanced BTC
FOENIX-CCA2	Futibatinib (FGFR2 inhibitor) in *FGFR2* fusion/rearrangement^155^	Phase 2	*FGFR2* fusion/rearranged iCCA	ORR 41.7%, median PFS 9.2 mo	Demonstrated efficacy of FGFR2-targeted therapy in genetically defined iCCA
ClarIDHy	Ivosidenib (IDH1 inhibitor) vs. placebo^82^	Phase 3	*IDH1*-mutant advanced cholangiocarcinoma	Improved PFS (2.7 vs. 1.4 mo)	Highlighted the role of targeted therapy in *IDH1*-mutant iCCA.
HERIZON BTC-01	Zanidatamab (HER2-targeted bispecific antibody)^70^	Phase 2	*HER2*-positive advanced BTC	ORR 40%, median PFS 5.5 mo	Highlighted HER2 as a therapeutic target in iCCA
NIFTY	Liposomal irinotecan+fluorouracil vs. fluorouracil alone^46^	Phase 2	Second-line BTC	Improved PFS (4.2 vs. 1.7 mo)	Supported the role of second-line therapy in advanced BTC

Abbreviations: BTC, biliary tract cancer; FGFR2, fibroblast growth factor receptor 2; iCCA, intrahepatic cholangiocarcinoma; IDH1, isocitrate dehydrogenase; ORR, objective response rate; OS, overall survival; PFS, progression-free survival.

## A NEW ERA OF TARGETED THERAPY

The rapid progress in gene detection technologies described above has significantly advanced the molecular understanding of iCCA, paving the way for precision oncology as a promising treatment paradigm. This approach enables the identification of subpopulations likely to benefit from targeted therapies, thereby personalizing treatment strategies and improving clinical outcomes.^48^,^49^,^50^ While precision oncology has significantly improved survival rates and prognoses in cancers such as breast and lung cancer,^51^,^52^ challenges remain. Many patients develop drug resistance, limiting the durability of these therapies.^53^ In iCCA, ~40%–50% of patients present with at least one actionable genetic alteration.^54^,^55^

### Receptor tyrosine kinase inhibitors

Receptor tyrosine kinases (RTKs) play a pivotal role in mediating critical signaling pathways associated with cancer progression. Abnormal RTK activation is a common feature observed in iCCA, contributing to tumor development and growth.^56^ Among RTKs, the fibroblast growth factor receptor (FGFR) family is particularly significant in driving tumor growth and angiogenesis.^57^,^58^,^59^ *FGFR2* mutations or fusions are found in 10%–15% of iCCA cases and play a key role in driving the initiation and progression of disease, making it a prime therapeutic target.^60^

Pemigatinib, approved in 2020 as the first pan-*FGFR* inhibitor specifically targeting *FGFR2* fusion-positive CCA, demonstrated promising efficacy in the FIGHT-202 trial. The trial reported an objective response rate (ORR) of 35.5%, a median PFS (mPFS) of 6.9 months, and a median OS of 21.1 months, highlighting its potential as a targeted therapy for this genetically defined subset of CCA.^61^ Futibatinib, another pan-*FGFR* inhibitor, demonstrated an ORR of 41.7% and a disease control rate (DCR) of 82.5% in patients with *FGFR2* fusion–positive iCCA. In the FOENIX-CCA2 trial, the reported median OS was 20 months.^62^ Additionally, a new selective *FGFR*-targeting multi-kinase inhibitor, tinengotinib, demonstrated a 94.7% DCR in *FGFR2* fusion/rearrangement CCA, including *FGFR*-resistant patients, during a phase 2 trial.^63^ Similarly, a dose escalation study involving lirafugratinib (RLY-4008), a highly selective oral *FGFR2* inhibitor, showed a DCR of 88% and 80% in FGFR inhibitor-naïve CCA and CCA with prior FGFR inhibitor use, respectively.^64^,^65^

Other promising RTK-targeted therapies include neurotrophic receptor tyrosine kinase (NTRK) inhibitors. Although *NTRK* fusions are rare in CCA, they are highly actionable. First-generation *NTRK* inhibitors such as larotrectinib and entrectinib have demonstrated remarkable ORRs ranging from 57% to 75% in *NTRK* fusion-positive cancers.^66^,^67^,^68^

*HER2* inhibitors also hold significant potential, as *HER2* amplification or overexpression is observed in 5%–20% of CCA cases. HER2-targeted therapies include trastuzumab plus pertuzumab, zanidatamab, trastuzumab deruxtecan, and tucatinib plus trastuzumab.^69^,^70^,^71^,^72^,^73^ Zanidatamab, a HER2-targeted bispecific antibody, has shown encouraging results in early trials with HER2-positive BTC. It achieved an ORR of 40%, with a median duration of response (DOR) of 8.1 months in patients with advanced BTC.^70^ Recently, zanidatamab received accelerated approval by the FDA based on its efficacy and potential to improve outcomes in this challenging cancer subtype, highlighting a significant advancement in HER2-targeted therapy for BTC.^74^,^75^ Trastuzumab deruxtecan has shown strong effectiveness in HER2-positive cancers, including breast, gastric, and non–small cell lung cancer (NSCLC).^76^,^77^,^78^ In the phase 2 HERB trial, 22 HER2-positive BTC patients achieved a 36% ORR, with mPFS of 5.1 months and OS of 7.1 months.^72^ The phase 2 DESTINY-PanTumor02 trial (41 BTC patients) reported an ORR of 22%, with higher HER2 expression (IHC 3+) showing a 56% ORR.^79^ This led to a tumor-agnostic FDA approval for this novel antibody–drug conjugate.

### Isocitrate dehydrogenase (IDH) inhibitors

Mutations in *IDH1* are present in ~10%–15% of iCCA cases, leading to the accumulation of 2-hydroxyglutarate, a metabolite implicated in driving tumorigenesis.^80^,^81^ Ivosidenib, an IDH1 inhibitor, has shown efficacy, as demonstrated in the ClarIDHy trial,^82^ where it significantly improved mPFS from 1.4 months to 2.7 months, resulting in FDA approval for use in *IDH1*-mutated CCA.^83^ Similarly, enasidenib, an IDH2 inhibitor already approved for leukemia, is under evaluation for its potential effectiveness in treating solid tumors, including CCA.^84^

### BRAF and KRAS inhibitors

*BRAF V600E* mutations, present in 3%–7% of iCCA cases, are effectively targeted by the combination of dabrafenib (a BRAF inhibitor) and trametinib (a MEK inhibitor). This approach achieved an impressive ORR of 51% in the ROAR trial, a phase 2 study. Notably, these drugs have also received tumor-agnostic FDA approval for *BRAF V600E*–mutant solid tumors, emphasizing their broad applicability and efficacy across multiple cancer types.^85^,^86^,^87^ Only a limited number of BTC patients have *KRAS G12C* mutations; however, encouraging responses were observed in adagrasib, an oral *KRAS G12C* tyrosine kinase inhibitor in the KRYSTAL-1 (NCT03785249) study and in sotorasib in the CodeBreaK 100 (NCT03600883) study. Furthermore, new *KRAS G12D* inhibitors, *KRAS G12V* inhibitors, and pan-RAS inhibitors are in development, offering the potential for targeting this more commonly found mutation in BTCs.^88^,^89^,^90^,^91^,^92^ This underscores the critical need to screen BTC patients for *KRAS* mutations to guide treatment with emerging therapies.^89^

### The significance of p53 mutations and the MDM2–p53 pathway in iCCA

The p53 protein is a critical tumor suppressor that plays a central role in regulating key cellular processes, including DNA repair, cell cycle arrest, and apoptosis.^93^,^94^,^95^ In iCCA, mutations in the *TP53* gene are observed in ~20%–30% of cases. These mutations compromise the ability of p53 to regulate cell growth and apoptosis, leading to unchecked cellular proliferation and contributing to tumor progression.^96^,^97^

The interaction between MDM2 and p53 further complicates the landscape of BTCs. While *MDM2* amplifications are rare in BTCs, when present, they play a significant role in tumor development by promoting excessive degradation of p53, thereby inactivating its tumor-suppressive functions.^96^ Targeting this pathway, inhibitors of the MDM2–p53 interaction, such as brigimadlin, have emerged as promising therapeutic agents. These inhibitors disrupt MDM2–p53 binding, stabilizing p53 and restoring its tumor-suppressive activity, with particular effectiveness in cancers harboring wild-type *TP53*.^98^ These advancements underscore the therapeutic potential of targeting the MDM2–p53 axis in iCCA and related malignancies.

Combination strategies are emerging as a promising approach in the treatment of BTCs. Preclinical and early-phase clinical trials have shown that MDM2 inhibitors exhibit enhanced efficacy when combined with chemotherapy or immune checkpoint inhibitors, offering new avenues for therapeutic advancements.^99^,^100^ Among these, idasanutlin (RG7388), an orally bioavailable MDM2 antagonist, has demonstrated potential by stabilizing p53 and inducing tumor regression.^24^ Similarly, milademetan (DS-3032) has shown notable activity in patients with *MDM2*-amplified tumors during phase 1 and 2 clinical trials.^101^ Brigimadlin (BI 907828), an oral MDM2–p53 antagonist (reactivates the tumor-suppressor function of p53 by inhibiting its interaction with MDM2, a protein amplified in 5%–8% of BTC cases), has demonstrated promising efficacy in phase 1a/2b trials. Among 12 patients, 50% achieved partial responses (PR), and the overall DCR was 83.3%. Brigimadlin showed durable responses and a manageable safety profile, with dose adjustments effectively addressing side effects.^98^,^102^ Although these results hinted towards the therapeutic potential for brimigadlin, all further evaluations with brimigadlin have been terminated owing to the discontinuation of the drug. The oral small molecule MDM2 inhibitor APG-115/alrizomadlin has shown promising antitumor activity among patients with p53 wild-type salivary gland tumors, and may represent an avenue for future exploration in BTCs.^103^

The integration of molecular profiling into routine clinical practice is critical for identifying patients most likely to benefit from these targeted therapies. Advances in companion diagnostic technologies, such as ctDNA analysis, are expected to play a transformative role in optimizing treatment selection and monitoring therapeutic responses, paving the way for more personalized and effective management strategies.^24^,^104^

### Other emerging therapeutic areas

Immune checkpoint inhibitors (ICIs) have also shown promise, with durvalumab yielding encouraging results in the TOPAZ-1 trial by significantly improving mPFS in patients with advanced disease.^41^ Combination ICI therapy with nivolumab plus ipilumab can be considered as both first-line and subsequent-line therapy (in ICI-naïve cases) for patients with tumor mutational burden-high (TMB-H; defined as TMB≥10 mutations per megabase) disease based on data from the CheckMate 848 study.^105^ Similarly, pembrolizumab can be considered for subsequent-line therapy in ICI-naïve TMB-H cases, although clinical trial data to support pembrolizumab use in this setting are limited.^20^,^106^ Furthermore, early research into chromatin remodeling inhibitors, including histone deacetylase (HDAC) inhibitors and methyltransferase inhibitors, is currently underway, highlighting innovative avenues for therapeutic development in BTC management.^3^,^21^

## FACTORS TO CONSIDER WHEN SELECTING SUBSEQUENT THERAPY

Selecting patients for targeted therapy in BTC requires integrating clinical and molecular data for optimal outcomes. Molecular profiling is crucial, involving MMR status evaluation via IHC or PCR to identify dMMR tumors, which respond to immune checkpoint inhibitors like pembrolizumab.^3^,^21^,^99^ HER2 status should be assessed using IHC, with ISH for equivocal cases, enabling HER2-directed therapies like trastuzumab plus pertuzumab, zanidatamab, trastuzumab deruxtecan, and tucatinib plus trastuzumab.^3^,^21^,^99^ Next-generation sequencing identifies actionable mutations such as *FGFR2* fusions, *IDH1* mutations, and *NTRK* alterations, critical for personalized therapy.^100^ In cases where sufficient tissue from biopsy or prior surgery is unavailable, ctDNA profiling is an alternative, with emerging evidence supporting its utility.^24^

Current liquid biopsy platforms have demonstrated limited sensitivity for detecting *FGFR2* fusions, potentially leading to missed diagnoses. Studies report that although liquid biopsy can identify *FGFR2* fusions in plasma, detection rates often fall short of optimal levels, ranging from 83% to 88.9% across various cohorts. This limitation is particularly significant in iCCA, where FGFR2 fusions are relatively common and serve as critical therapeutic targets.^29^,^107^,^108^

Given these limitations, tissue biopsy remains the gold standard for detecting *FGFR2* fusions, offering superior accuracy. Comprehensive genomic profiling of tissue samples consistently achieves higher detection rates; for example, tissue biopsies have identified *FGFR2* rearrangements in up to 9% of iCCA cases, compared to lower rates seen with plasma-based assays.^109^,^110^

In iCCA, repeat tissue biopsies should be considered when initial samples are insufficient or inconclusive. Since iCCA tumors are generally accessible for biopsy, repeating the procedure is often feasible and advantageous for ensuring accurate molecular characterization. Considering the high prevalence and actionable nature of *FGFR2* fusions in iCCA, obtaining adequate tissue material is essential for guiding targeted therapy decisions.^111^

In summary, while liquid biopsy offers a non-invasive option when tissue is unavailable, tissue biopsy remains the preferred method for *FGFR2* fusion detection due to its greater sensitivity and reliability. Repeat biopsies should be strongly considered in iCCA to achieve comprehensive genomic profiling and optimize personalized treatment strategies.

A multidisciplinary team ensures personalized therapy based on molecular findings, patient-specific factors, and systemic considerations. Eligibility for targeted therapy requires an Eastern Cooperative Oncology Group (ECOG) performance status (PS) of 0-1 and preserved liver function.^21^

In clinical practice, eligibility for targeted therapies, including pembrolizumab, is generally guided by an ECOG PS of 0–1, as demonstrated in the KEYNOTE-966 trial.^42^ However, emerging evidence suggests that patients with higher ECOG PS scores, such as 2 or 3, may still derive benefit from immunotherapy under specific circumstances.

For instance, a study by Pietrantonio et al^112^ reported that patients with MSI-H cancers and ECOG PS 2 or 3 could still achieve meaningful responses to immune checkpoint inhibitors like pembrolizumab. In their cohort, 33% of patients with MSI-H tumors and ECOG PS 2 or 3 experienced a response, with a median duration of response of 16.9 months.^112^ These findings suggest that pembrolizumab could be a therapeutic option for patients with ECOG PS 2 or 3 and MSI-H tumors, even though such patients are often excluded from clinical trials.

Thus, while standard eligibility criteria for pembrolizumab typically require an ECOG PS of 0–1, carefully selected patients with ECOG PS 2 or 3 and MSI-H status may still be considered for treatment based on individualized clinical judgment and emerging data. This approach should be undertaken with the integration of palliative care measures and close monitoring for potential adverse effects.^112^

Treatments are prioritized using the ESMO Scale for Clinical Actionability of Molecular Targets (ESCAT), focusing on high-utility targets, and are often applied as second-line therapies after gemcitabine–cisplatin progression.^21^,^39^,^113^

Subsequent therapies depend on the presence of molecular alterations like *FGFR2* fusions and *IDH1* mutations. Treatment decisions also consider patient-specific factors, prior therapy response, and disease progression.^114^,^115^ Adherence to evidence-based guidelines and access to molecular testing and clinical trials are essential for effective care.^115^

## THERAPEUTIC CHALLENGES IN ICCA

Despite advances in treatment, several challenges persist in the management of iCCA. Drug resistance remains a significant hurdle, as resistance to targeted therapies can limit their long-term efficacy. For example, secondary mutations in *FGFR2* can reduce the effectiveness of FGFR inhibitors, posing a challenge to sustained therapeutic success.^116^

Additionally, the heterogeneity of genetic alterations in iCCA complicates the identification and selection of optimal therapies, as the diverse molecular landscape requires precise and individualized treatment approaches.^55^,^56^ Moreover, limited access to molecular profiling, especially in low-resource settings, further exacerbates these challenges. The lack of availability and affordability of comprehensive genomic profiling restricts the ability to identify actionable mutations, thereby hindering the implementation of precision oncology.^49^ These barriers underscore the need for continued innovation and equitable access to advanced diagnostic and therapeutic tools.

## FUTURE DIRECTIONS IN SYSTEMIC THERAPY MANAGEMENT

On the therapeutic front, research is actively exploring the potential of immunotherapy combinations. Combinations of immune checkpoint inhibitors with other agents, cellular therapy approaches, and novel vaccines all aim to enhance therapeutic efficacy and overcome challenges related to immune resistance. These approaches represent a new frontier in iCCA treatment, highlighting the potential for personalized and more effective care.^21^ Future directions in the management of iCCA focus on innovative diagnostic and therapeutic strategies to improve outcomes. One promising approach remains the use of ctDNA as a diagnostic tool, which offers a minimally invasive method to monitor tumor dynamics, identify actionable genetic mutations, and track the development of treatment resistance. This technique serves as an effective alternative for patients who are unable to undergo traditional tumor biopsies, expanding diagnostic capabilities.^113^,^117^

## REDEFINING CURATIVE OPTIONS

In current practice, the only curative treatment for iCCA is surgical resection with negative margins (R0 resection); however, as described previously, most patients with iCCA present with unresectable disease.^3^,^12^ Additionally, many patients with iCCA may not be candidates for surgical resection due to advanced disease, comorbidities, underlying chronic liver disease, or portal hypertension, which may occur alone or in combination with each other.^118^,^119^ Liver transplantation (LT) offers the prospect of R0 resection for eligible patients with the removal of all intrahepatic lesions, which may or may not be visible on imaging.^120^

Although data are limited, previous studies have evaluated the role of LT in patients with advanced or unresectable iCCA. A multicenter, international retrospective study reported outcomes of 81 patients who underwent LT and were incidentally found to have iCCA at explant pathological examination.^121^ In a subgroup analysis, those patients who had “very early” iCCA (single tumor <2 cm) had decreased risk of recurrence and increased OS compared to the advanced group (single tumor >2 cm or multifocality).^121^ The role of systemic therapy utilized alongside LT for patients with iCCA has also been explored. In a single institutional prospective study, patients with unresectable iCCA were considered eligible for LT if their disease showed “stability” for 6 months on systemic therapy.^122^ Initial results were reported from the first 12 patients enrolled in the study, and 6 patients among these were deemed eligible for transplantation. Across a median follow-up of 36 (IQR: 29–51) months post-transplantation, 3 patients developed recurrent disease.^122^ Subsequent long-term outcomes from the same study were also reported, with 37 patients being listed for LT, out of which 18 eventually underwent LT.^123^ Notably, most patients (12/18, 66.7%) in the cohort that underwent transplantation had no pre-existing liver disease. Across a median follow-up of 26 months, 1-year, 3-year, and 5-year OS rates in patients who underwent LT were 100%, 71%, and 57%, respectively.^123^ Overall survival was significantly prolonged among patients who underwent LT *(p=*0.002).^123^ Although limited by the modest sample size and duration of follow-up, there were no patients alive at 2 years in the group that did not undergo LT.^123^ Future studies exploring the role of LT itself in iCCA patients both with and without cirrhosis, along with a focus on optimizing perioperative systemic therapy, are imperative to help redefine this potentially curative treatment strategy in patients with surgically unresectable disease.^124^

Notably, the Organ Procurement and Transplantation Network (OPTN) has recently proposed a MELD exception for patients with unresectable solitary iCCA.^125^ The proposal suggests MELD exception points for iCCA ≤3 cm that demonstrates 6 months of tumor stability following locoregional or systemic therapy. Additionally, transplant candidates would be eligible for MELD exception extension if their tumors remain smaller than 3 cm and confined to the liver as confirmed by quarterly imaging. The proposed MELD exception score would be set at 3 points less than the Median MELD at Transplant (MMaT-3).^125^

Locoregional therapies (LRT) also offer a promising avenue for future exploration in patients with unresectable iCCA.^126^ Certain modalities of LRT may offer superior outcomes as compared to others, as shown in a retrospective comparative analysis of 71 patients with iCCA from an Italian multicenter cohort. High-powered microwave ablation (MWA) (n=35) was found to be superior to radiofrequency ablation (RFA) (n=36) in terms of disease-free survival (*p*<0.005), PFS *(p<*0.005), and OS (adjusted *p*=0.001).^127^ Yan et al^128^ showed that combining chemotherapy with RFA/MWA thermal ablative therapy in unresectable, untreated iCCA significantly improved median OS as compared to chemotherapy alone (15.23 vs. 7.97 mo; *p*=0.009). Transarterial interventions, namely selective internal radiation therapy (SIRT)/transarterial radioembolization (TARE), transarterial chemoembolization (TACE), and hepatic arterial infusion (HAI), are considered safe and technically feasible for patients with iCCA. Their role in unresectable iCCA has been evaluated but warrants further exploration.^3^,^129^,^130^,^131^,^132^ In a study which included 319 patients with multifocal iCCA and compared HAI with floxuridine chemotherapy versus resection, similar median OS was observed (20.3 mo vs. 18.9 mo; *p*=0.32).^133^ Notably, the HAI group demonstrated significantly lower 30-day postoperative mortality (0.8% vs. 6.2%, *p*=0.01).^133^ Along these lines, a retrospective study of 268 patients compared HAI chemotherapy (with or without systemic chemotherapy) versus GemCis in patients with liver-confined unresectable iCCA and significantly improved OS was noted with HAI (27.7 vs. 11.8 mo, *p*<0.001).^134^ Similar to RFA/WMA, studies evaluating combination strategies with transarterial approaches and systemic therapy have shown promise, both in terms of downstaging of tumors as well as overall survival.^135^,^136^,^137^ To explore the utility of LRTs as potentially curative treatment options for patients with iCCA, future investigations must explore comparisons of LRT modalities, along with the utility of LRT used in addition to systemic therapy for patients with unresectable iCCA.

## ROLE OF PATIENT ADVOCACY: WITH PERSPECTIVES OF A PATIENT ADVOCATE

For patients with BTCs, advocacy is often considered essential to help navigate the complexities of diagnosis, treatment, and support.^138^ Patient advocates not only provide education, resources, and opportunities for those navigating the cancer care continuum, but they also prioritize the individual and provide emotional support and guidance. Patient advocates recognize the power of education, empowerment, and community in guiding individuals through the complex landscape of BTC diagnosis and treatment.^138^

Early diagnosis and intervention often depend on awareness campaigns led by advocacy groups, and the importance of patient advocacy in the setup of early detection programs for cancer has been recognized.^139^ Recognizing symptoms like jaundice, unexplained weight loss, or abdominal pain and encouraging patients to seek timely medical attention can be associated with life-saving differences.^140^ Earlier detection, diagnosis, and intervention can be promoted through awareness campaigns led by advocacy groups.^140^,^141^ Advocacy also aims to promote equitable access to molecular testing, which can help ensure that treatment plans align with patients’ biological and psychosocial interests.^138^

Patient advocates are skilled at bridging the gap between patients and the medical community, fostering better communication and understanding. Through organizations like the Cholangiocarcinoma Foundation, patients receive educational resources, care kits to provide comfort for symptoms associated with treatment, support through personal guidance with those who have been in the same position, volunteer opportunities that bring a sense of accomplishment, and access to a global support network.^142^ In the setting of healthcare discussions, members of the healthcare team such as nurses often take on the role of patient advocates in multidisciplinary meetings.^143^ Advocating for the emotional and mental well-being of patients is particularly important.^144^ Resources such as the Cholangiocarcinoma Foundation allows for the patient and their support system to have access to a community that provides patient and caregiver resources, peer support networks, counseling, as well as educational materials that are easy to access and understand.^142^ It is vital for members of healthcare teams to provide the resources and encouragement necessary for patients to be active advocates for themselves.

Another crucial role played by patient advocates is encouraging patient participation in clinical trials.^145^ Patient advocates emphasize the rigorous ethical standards governing trials by addressing fears and misconceptions, such as concerns about being “guinea pigs.”^142^ Through these advocacy programs, patients can receive close monitoring and access to multidisciplinary care teams during trials, with increased access to novel therapies.^142^ Patient advocacy has also been shown to be beneficial for patient enrollment in technically challenging clinical trials.^146^

In summary, patient advocacy transforms the BTC experience by offering hope, education, and a collective voice for change. It can inspire trust in emerging science while ensuring that patients remain central to advancements in treatment and care.

## ROLE OF A MULTIDISCIPLINARY TEAM

Akin to other malignancies, BTCs, including iCCA, require a multidisciplinary approach to optimize clinical outcomes.^147^,^148^,^149^ Expertise from multiple disciplines allows for the treatment needs of patients with iCCA to be addressed in different ways and at different times throughout their cancer journey.^150^ As per the European Network for the Study of Cholangiocarcinoma, an ideal multidisciplinary team for CCAs consists of an oncologist, surgeon, diagnostic and interventional radiologist, hepatologist, pathologist, and gastroenterologist.^147^ The presence of a palliative care physician, nurse, dietitian, translational researcher, psychologist, and social worker is also recommended.^147^

From the time of diagnosis, medical oncologists, hepatobiliary surgeons, radiologists, pathologists, and gastroenterologists collaborate to provide an accurate diagnosis and determine the extent of disease involvement.^151^ Imaging studies and endoscopic procedures such as endoscopic ultrasound and endoscopic retrograde cholangiopancreatography are often used to accurately ascertain the extent of disease.^151^ After the initial diagnosis is received, surgical options, (neo)adjuvant chemotherapy, targeted therapies, and locoregional therapies should be discussed in a multidisciplinary tumor board.^152^,^153^

Biliary tract cancers are often diagnosed at a late stage which can mean high symptom burden in these patients.^12^ Managing pain, jaundice, fatigue, and weight loss in these patients is complex and requires care from both nurses and palliative care physicians.^143^ Nurses also help to manage treatment-related side effects and provide education on supportive care medications.^143^ In our experience, nurse coordinators or navigators often serve as a point of contact for the patient and their family members. They help to facilitate communication among the multidisciplinary team and ensure the treatment plan is expedited in a timely manner. Palliative care teams also provide psychological and spiritual support when patients are nearing end-of-life decisions.^154^ Social workers can help facilitate logistical issues, such as transportation or financial issues.^147^

Multidisciplinary teams foster collaboration and provide a holistic approach in managing the care of iCCA patients. Ensuring the medical, psychological, and emotional needs of patients are met provides a comprehensive care model that can enhance clinical outcomes and the overall patient care experience.

## ROLE OF CLINICAL TRIALS: A PATIENT AND PATIENT ADVOCATE’S PERSPECTIVE

Clinical trials represent the forefront of progress in BTC research and treatment, offering hope, improved survival rates, and enhanced quality of life. As a long-term cholangiocarcinoma survivor, I [Melinda Bachini] am here today because of a clinical trial. This experience emphasizes their importance and the need to provide an understanding of clinical trials for patients and clinicians alike. Biliary tract cancer clinical trials have evolved significantly over the past decade. Today, ~ 83 BTC trials are available in the United States alone, representing a significant expansion of opportunities to explore progressive therapies. However, significant gaps remain, particularly in the second-line setting for patients without targetable mutations and in the development of neoadjuvant therapies aimed at reducing recurrence rates post-surgery. Addressing these unmet needs could revolutionize BTC care and provide more viable options for patients facing limited treatment options.

Participation in clinical trials should be considered soon after a patient’s diagnosis, yet patients are too often not offered these options. Misunderstandings about placebos, fear, and logistical and financial challenges deter too many from considering trials. It is vital to dispel these myths. Clinical trials rarely involve placebos without a standard-of-care treatment arm, ensuring patients receive the best care. Far from being a last resort, trials offer access to cutting-edge therapies that may otherwise be unavailable, which have the potential for better outcomes and longer survival.

As trusted sources, clinicians should be prepared to emphasize the unique benefits of trials to newly diagnosed patients. Trial participants receive closer monitoring and access to a multidisciplinary care team and have a special role in contributing to medical advancements. Studies have shown that patients who participate in trials report higher confidence in their care and greater satisfaction with their treatment journey. Despite these advantages, awareness of and access to trials remain a challenge. Expanding access requires fostering international collaboration, exploring innovative combination therapies, and prioritizing patient-centric designs that align with the real-world needs of those living with BTC. Partnerships among researchers, industry leaders, and patient advocacy groups are essential in creating trial opportunities that are accessible, easy to communicate to patients, and equitable.

Biliary tract cancer is a rare and aggressive cancer, often diagnosed late, limiting treatment options. Clinical trials offer hope and innovation for patients like me, who once faced a bleak prognosis. By participating in a trial, I gained access to an emerging therapy that saved my life and contributed to the scientific community’s understanding of new therapies. The global BTC community must work together to accelerate progress, reduce barriers, and ensure that clinical trials are not only an option but a foundation of treatment strategies. These efforts are critical for advancing research, improving survival rates, and, ultimately, finding a cure. With continued focus, support, and collaboration, we can create a future where no one faces BTC without options or hope.

## CONCLUSIONS

Intrahepatic cholangiocarcinoma is a growing oncologic challenge where the number of patients eligible for curative intent surgical resection remains limited. The non-specific clinical presentation makes early diagnosis challenging, and increased awareness is needed. Many patients with iCCA have therapeutically targetable alterations detected through genomic profiling of their tumors, and molecular testing is an integral part of the clinical management of iCCA. Several patient, disease, and clinical factors, including tumor molecular profile, response to prior therapy, performance status, and patient preferences, must all be taken into consideration using a multidisciplinary approach during therapeutic planning in patients with iCCA. Single-center studies have shown the potential benefit of liver transplantation alongside systemic therapy for patients with unresectable disease, and the exploration of these strategies as curative intent treatments is warranted. Perspectives from patients and patient advocates highlight the importance of advocacy and access to clinical trial advances in delivering patient-centered care.
